# A genome-wide transcriptional profiling of sporulating *Bacillus subtilis* strain lacking PrpE protein phosphatase

**DOI:** 10.1007/s00438-013-0763-7

**Published:** 2013-07-04

**Authors:** Adam Iwanicki, Krzysztof Hinc, Anna Ronowicz, Arkadiusz Piotrowski, Aleksandra Wołoszyk, Michał Obuchowski

**Affiliations:** 1 Laboratory of Molecular Bacteriology, Intercollegiate Faculty of Biotechnology, University of Gdańsk and Medical University of Gdańsk, Dębinki 1, 80-211 Gdańsk, Poland; 2 Department of Biology and Pharmaceutical Botany, Medical University of Gdańsk, Hallera 107, 80-416 Gdańsk, Poland

**Keywords:** *Bacillus subtilis*, Sporulation, PrpE protein phosphatase, Microarray, Transcriptional profiling

## Abstract

The sporulation process is a complex genetic developmental program leading to profound changes in global gene expression profile. In this work, we have applied genome-wide microarray approach for transcriptional profiling of *Bacillus subtilis* strain lacking a gene coding for PrpE protein phosphatase. This protein was previously shown to be involved in the regulation of germination of *B. subtilis* spores. Moreover, the deletion of *prpE* gene resulted in changing the resistance properties of spores. Our results provide genome-wide insight into the influence of this protein phosphatase on the physiology of *B. subtilis* cells. Although the precise role of PrpE in shaping the observed phenotype of Δ*prpE* mutant strain still remains beyond the understanding, our experiments brought observations of possible indirect implication of this protein in the regulation of cell motility and chemotaxis, as well as the development of competence. Surprisingly, *prpE*-deleted cells showed elevated level of general stress response, which turned out to be growth medium specific.

**Electronic supplementary material:**

The online version of this article (doi:10.1007/s00438-013-0763-7) contains supplementary material, which is available to authorized users.

## Introduction

*Bacillus subtilis* is able to form endospores in response to unfavorable environmental conditions. In the process of sporulation, bacterial cell undergoes asymmetric division resulting in the formation and subsequent release of highly durable spore. Sporulation is a complicated developmental process involving profound changes in gene expression profile. These changes are controlled by a cascade of σ factors of RNA polymerase as well as various transcription factors. Main transcription factor responsible for entry into sporulation is Spo0A (Fawcett et al. 2000; Molle et al. 2003). The activity of this protein is regulated through phosphorylation by phosphorelay that integrates environmental and physiological signals in decision to sporulate (Jijang et al. 2000). In its phosphorylated state, Spo0A targets genes involved in the formation of a polar septum (Levin and Losick 1996) as well as genes driving activation of first two compartment-specific sigma factors of RNA polymerase, σ^E^ and σ^F^. The σ^F^ factor is activated only in the forespore compartment and σ^E^ in the mother cell. The activation of σ^F^ is critical for all subsequent spore development and is regulated by a complex mechanism (Hilbert and Piggot 2004). As the process of sporulation progresses these sigma factors become replaced by σ^G^ (forespore) and σ^K^ (mother cell) (Stragier and Losick 1996).

The mature spore is highly resistant to harsh environmental conditions (Nicholson et al. 2000; Takamatsu and Watabe 2002). It can remain dormant for very long periods of time and re-initiate vegetative growth in the process of germination once the environment becomes growth favorable. Spore can constantly monitor the environment and respond to the availability of nutrients or other germination factors (Moir et al. 2002; Setlow et al. 2003; Shah et al. 2008). Breaking of spore dormancy depends on different pathways depending on the conditions. In case of some germinants, for example l-alanine or an AGFK mixture (asparagine, glucose, fructose, potassium), spore germination depends on specific germination receptors encoded by three operons: *gerA*, *gerB* and *gerK* (Corfe et al. 1994a, b; Moir et al. 1994; Zuberi et al. 1987). In case of other germination inducing factors the receptors remain undiscovered. Expression of genes coding for germination receptors takes place only in the forespore compartment and reaches a maximum about 4–6 h upon initiation of sporulation (Corfe et al. 1994b; Feavers et al. 1990).

The PrpE protein phosphatase has been shown to influence the process of spore germination in *B. subtilis*. Spores of a strain deleted for the *prpE* gene are not able to initiate germination in response to l-alanine. The expression of the *gerA* gene coding for germination receptor was originally shown to be significantly lowered in this strain. Moreover, spores of Δ*prpE* strain are less resistant for desiccation as compared to the wild-type strain (Hinc et al. 2006). All these data point to the role of PrpE in regulation of spore formation as well as germination. It is worth adding, that the analysis of amino acid sequence of the PrpE protein shows the presence of motifs characteristic for protein phosphatases of PPP family as well as diadenosine polyphosphate hydrolases. Both activities of PrpE have been experimentally shown in vitro (Iwanicki et al. 2002).

Analysis of global changes in gene expression profiles has become a routine technique in molecular biology. This also accounts for microbiology, where DNA microarrays covering all annotated genes of various bacteria are commercially available. The progress of microarray technology is reflected in the number of works published involving the usage of this technique. *B. subtilis* has also been subjected to transcription profiling in various conditions such as sporulation (Fawcett et al. 2000), germination (Keijser et al. 2007), cold-shock response (Kaan et al. 2002) or phosphate limitation (Botella et al. 2011).

In this work, we have performed a transcriptional analysis of sporulating Δ*prpE* strain. We used a DNA microarray technique to compare transcriptional profiles of wild-type 168 strain and Δ*prpE* strain during sporulation. The results showed that over 490 genes were significantly upregulated in Δ*prpE* strain, while another 220 genes showed downregulation. Most of upregulated genes belong to the general stress sigma factor regulon and σ^D^ regulon responsible for motility and chemotaxis. The group of significantly downregulated genes was not as homogenous but we have found that the part of SinR regulon encompassing genes responsible for the production of exopolysaccharide showed lower expression level in Δ*prpE* strain. Moreover, we have found that the elevated level of the σ^B^ regulon expression is media dependent.

## Materials and methods

### Bacterial strains

*Bacillus subtilis* 168 (Anagnostopoulos and Crawford 1961) was used as a wild-type strain. Strain lacking PrpE phosphatase (Δ*prpE*) was originally constructed in 168 genetic background (Iwanicki et al. 2002). A strain 2458 with genetic background of 168 harboring *spoIIQ* promoter fused to GFP encoding gene in non-essential locus *amyE* was a kind gift from prof. Ezio Ricca. Chromosomal DNA of this strain was used to transform Δ*prpE* strain resulting in a strain BKH106 with following genotype Δ*prpE amyE::p*_*spoIIQ*_-*gfp cm* ^r^. A strain PB198 with genetic background of 168 harboring *ctc*-*lacZ* fusion in *amyE* locus was obtained from prof. Chester Price (Boylan et al. 1993). A strain BAW01 was constructed by transformation of strain Δ*prpE* with chromosomal DNA of PB198 resulting in Δ*prpE amyE::ctc*-*lacZ cm* ^r^ genotype.

### Sporulation conditions

Sporulation was induced using the resuspension method (Harwood and Cutting 1990). Cells were grown overnight in Sterlini–Mandelstam growth medium. On the next day, cultures were refreshed in fresh growth medium at an OD_600_ = 0.05 and grown at 37 °C until they reached an OD_600_ = 0.6. Cultures were centrifuged at 5,000×*g* for 5 min and resuspended in equal volumes of warm resuspension medium. The point of resuspension was defined as *T*_0_.

### RNA isolation, labeling, hybridization and scanning

RNA was isolated from sporulating bacteria of wild-type 168 and Δ*prpE* strains collected at indicated time points. 10 OD_600_ units of cultures were centrifuged at 5,000×*g* for 5 min and pellets were stored at −80 °C. Isolation of RNA was performed using Universal RNA Purification Kit (Eurx Ltd., Gdańsk, Poland) according to the manufacturer’s protocol with following modification. Prior to extraction, bacterial pellet was resuspended in 300 μl of TE buffer prepared on DEPC-treated water. 100 mg of acid-washed glass beads (Sigma, USA) were added and the suspension was vortexed in Mini-BeadBeater (Biospect Products Inc., Bartlesville, OK, USA) ten times for 30 s with 1 min of incubations on ice between the pulses. Next, the mixture was centrifuged 12,000×*g* for 10 min and 100 μl of supernatant was used for RNA extraction. DNA was removed by on-column DNase digestion with RNase-free DNase I (Thermo Fisher Scientific Inc., Waltham, MA, USA) as indicated in the manufacturer’s protocol. The concentration and purity of the extracted RNA was assessed using NanoDrop ND-1000 spectrophotometer (Thermo Fisher Scientific) and the quality was checked by gel electrophoresis. 1 μg of total RNA was used for the synthesis of double-stranded cDNA using Roche cDNA Synthesis System kit (Roche, Basel, Switzerland) using random hexamers (Thermo Fisher Scientific) as primers in the reverse transcription reaction. 250 ng of double-stranded cDNA was labeled using Roche Dual-Color DNA Labeling kit (Roche), 168 strain—Cy5, Δ*prpE*—Cy3). Hybridization was performed using NimbleGen 4x72k *B. subtilis* subsp. *subtilis* strain 168 NC_000964 microarray slides following standard operating protocol (Roche NimbleGen Inc.). Each slide consisted of four separate hybridization fields covering whole transcriptome of *B. subtilis* with eight 60-nucleotide probes per gene in two technical replicates. Scanning was performed using Roche MS 200 Microarray Scanner following standard operating protocol (Roche). Three independent biological replicates have been performed for each strain.

### Data analysis

The raw data (.pair file) were subjected to RMA (robust multi-array analysis) (Irizarry et al. 2003), including quantile normalization (Bolstad et al. 2003), and background correction as implemented in the NimbleScan software package, version 2.4.27 (Roche NimbleGen, Inc.). Normalized data were stored as Microsoft Excel spreadsheets. The SAM analysis (significance analysis of microarrays, Tusher et al. 2001) was performed with MeV package (Saeed et al. 2003) using data from three biological replicates adjusting delta parameter to keep the percentage of falsely positive genes below 1 %. The data were then averaged and used for further analyses. Fisher exact tests were performed with statistical R Package (R Development Core Team 2011). The hierarchical clustering and *K*-means clustering was performed using MeV package. The raw and normalized data were deposited at GEO (NCBI) under accession GSE44125.

### β-Galactosidase measurements

Cultures were grown at 37 °C with shaking. As a reach medium Difco nutrient broth (BD, Sparks, MD, USA) supplemented with 1 mM Ca(NO_3_)_2_, 10 μM MnCl_2_ and 1 μM FeSO_4_ was used. Samples were taken at indicated time points and stored at −20 °C until enzyme assays were carried out. After thawing, bacterial pellets were suspended in buffer Z (60 mM Na_2_HPO_4_, 40 mM NaH_2_PO_4_, 10 mM KCl, 1 mM MgSO_4_) containing 1 mM dithiothreitol (DTT), and 1/100 volume of lysis solution (1 mg/ml DNase, 10 mg/ml lysozyme) was added. The mixture containing lysed cells was incubated for 20 min at 37 °C and then centrifuged for 10 min at 10,000×*g* and 4 °C to remove the cell debris. The supernatant was used for measurement of protein concentration and β-galactosidase activity. Protein concentrations were measured using the Bradford reagent (Bio-Rad) as recommended by the manufacturer. Supernatants (200 μl) were mixed with 600 μl of buffer Z containing 1 mM DTT. Samples were placed in a 37 °C water bath, and 200 μl of *o*-nitrophenyl-β-d-galactopyranoside (4 mg/ml) was added. The reaction was stopped by addition of 500 μl of 1 M Na_2_CO_3_, and the absorbance was measured at 420 nm. β-galactosidase activity, in nmol of *o*-nitrophenol produced min^−1^ mg^−1^, was calculated using the following formula: (absorbance at 420 nm × 1.5)/(concentration of protein in mg/ml × volume of sample in ml × reaction time in min × 0.00486).

## Results

### Wild-type and Δ*prpE* strains initiate sporulation process at the same time

**Fig. 1:**
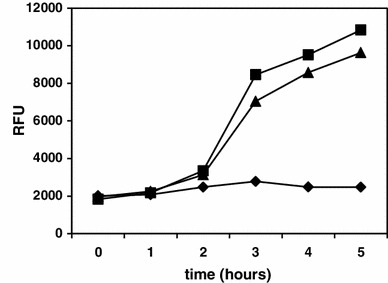
The activity of *p*_*spoIIQ*_ promoter as assessed by measurement of GFP fluorescence. Bacteria were grown in Sterlini–Mandelstam medium and the fluorescence of GFP was measured at indicated times. *Squares* indicate strain 2458 (*amyE::p*_*spoIIQ*_-*gfp*), *triangles* strain BKH106 (Δ*prpE* *amyE::p*_*spoIIQ*_-*gfp*) and *diamonds* strain 168. *Vertical axis values* indicate relative fluorescence units, *horizontal axis values* time in hours upon induction of sporulation

Bacterial monocultures show a large degree of diversity by heterogeneous cell differentiation and population subculturing. Stochastic population splitting has been demonstrated for variety of stress responses including sporulation, competence or motility (Dubnau and Losick 2006; Smits et al. 2006). The heterogeneity in *B. subtilis* spore formation has been suggested to be a result of heterochronic phosphorelay gene expression (de Jong et al. 2010). We wanted to analyze whether deletion of the *prpE* gene influences timing of sporulation initiation. To check that the sporulation process in Δ*prpE* strain initiates at the same time as in wild-type 168 strain, we have used a strain harboring σ^F^-dependent promoter of *spoIIQ* gene fused to *gfp* gene enabling for monitoring the progress of sporulation. The sporulation was induced as described in “Materials and methods” and the fluorescence of GFP protein was monitored. The results (Fig. 1) clearly indicate that both strains initiate sporulation at the same time.

### Transcriptional profiling of sporulating Δ*prpE* strain

Experiments were carried out with RNA from wild-type 168 cells and from cells mutant for PrpE. Cells were harvested 60, 130, 200, 270, 340 and 410 min upon initiation of sporulation, which roughly corresponds to sporulation times *T*_1_, *T*_2_, *T*_3_, *T*_4.5_, *T*_5.5_ and *T*_7_. Isolated total RNA was reverse transcribed to cDNA and labeled. Next, labeled cDNAs from each time point were hybridized against DNA microarrays covering all annotated genes of *B. subtilis*. Scanned fluorescence values were processed as described in “Materials and methods”. As a result, we obtained a panel of mutant to wild-type fluorescence ratios for each of time points. Upon the SAM analysis, we obtained 490 genes, which were significantly upregulated and 223 genes, which were significantly downregulated at least at one time point in Δ*prpE* strain as compared to wild-type strain. These numbers represent 11.9 and 5.4 % of all *B. subtilis* genes. The exact numbers of genes significantly up- and downregulated in Δ*prpE* as compared to the wild-type strain at each time point tested are listed in Table 1 and the distributions of Δ*prpE*/wild-type gene expression ratios are depicted as MA plots in Figure S1.

**Table 1:** The numbers of genes with statistically significant difference in expression between Δ*prpE* mutant and the wild-type strain as assessed by SAM analysis

	60 min	130 min	200 min	270 min	340 min	410 min
Upregulated	145	88	490	160	44	206
Downregulated	18	6	223	89	19	51

### Functional analysis

**Fig. 2:**
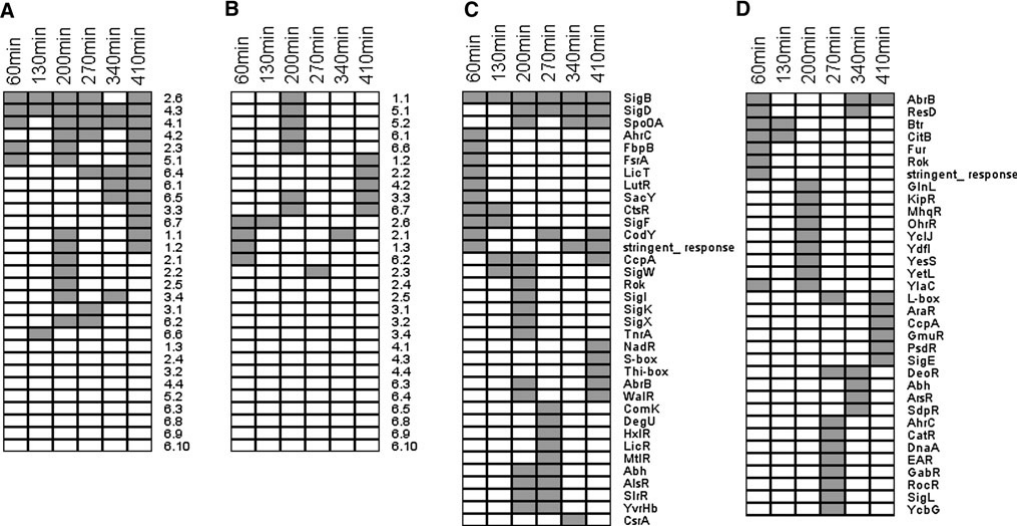
Hierarchical clustering of overrepresented functional categories of gene products (**a**, **b**) and regulons (**c**, **d**) (Mäder et al. 2012). **a** and **c** Upregulated genes, **b** and **d** downregulated genes. The categories are as follows: 1.1 cell wall and cell division, 1.2 transporters, 1.3 homeostasis, 2.1 electron transport and ATP synthesis, 2.2 carbon metabolism, 2.3 amino acid/ nitrogen metabolism, 2.4 lipid metabolism, 2.5 nucleotide metabolism, 2.6 additional metabolic pathways, 3.1 genetics, 3.2 RNA synthesis and degradation, 3.3 protein synthesis, modification and degradation, 3.4 regulation of gene expression, 4.1 exponential and early post-exponential lifestyles, 4.2 sporulation and germination, 4.3 coping with stress, 4.4 lifestyles/ miscellaneous, 5.1 prophages, 5.2 mobile genetic elements, 6.1 essential genes, 6.2 membrane proteins, 6.3 GTP-binding proteins, 6.4 phosphoproteins, 6.5 universally conserved proteins, 6.6 poorly characterized/putative enzymes, 6.7 proteins of unknown function, 6.8 short peptides, 6.9 ncRNA, 6.10 pseudogenes

Up- and downregulated genes were assigned their functional categories following classification proposed by Mäder et al. (2012). Based on this assignment, we assessed the overrepresentation of each functional category using Fisher exact test (α = 0.05) applied on calculated gene expression ratios between Δ*prpE* and wild-type strains. The individual functional categories were then subjected to hierarchical clustering using the technique of Eisen et al. (1998) (Fig. 2a, b). Following the same procedure genes were assigned to the regulons of transcription factors (Mäder et al. 2012) and the overrepresentation of each regulon was tested (Fig. 2c, d).

### Expression of sporulation RNA polymerase σ factors regulons

**Fig. 3:**
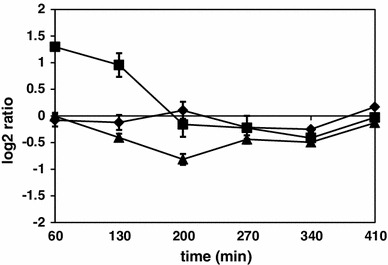
Average Δ*prpE*/168 gene expression ratios of germination receptors operons. *Diamonds* GerA operon, *squares* GerB operon, *triangles* GerK. *Vertical axis values* indicate normalized log_2_ of ratios, *horizontal axis values* time points upon induction of sporulation. *Error bars* indicate standard deviation of gene expression ratios

The *prpE*-deleted strain was shown to be unable to initiate germination of spores in response to some germination factors. Moreover, spores of Δ*prpE* strain are significantly less resistant to dry heat treatment (Hinc et al. 2006). These observations prompted us to analyze the expression ratio patterns of genes belonging to regulons of sporulation σ factors of RNA polymerase. Lists of genes under control of σ^F^, σ^E^, σ^G^ and σ^K^ were taken from Subtiwiki database (Mäder et al. 2012). The numbers of genes significantly up- and downregulated as assessed by SAM analysis listed in Table 2 indicated that the expression of only a minor percentage of these genes differs between the wild-type and Δ*prpE* strains. This observation was more evident when average gene expression ratios of Δ*prpE*/wild-type strain across all time points tested were represented as centroid graphs (Figure S2). We have additionally focused on the expression of *gerA*, *gerB* and *gerK* operons encoding germination receptors. A centroid graph of averaged Δ*prpE*/wild-type expression ratios of genes forming each operon (Fig. 3) revealed that the level of expression of *gerA* operon does not differ between both strains. The expression of *gerB* operon is elevated in Δ*prpE* mutant as compared to the wild-type strain at 60 and 130-min time points and these differences are statistically significant. In case of *gerK* operon, although the expression in Δ*prpE* strain seems to be lowered as compared to the wild-type strain, the difference is statistically significant only for *gerKB* and *gerKC* genes at 200-min time point.

**Table 2:** The numbers of genes with statistically significant difference in expression between Δ*prpE* mutant and the wild-type strain belonging to following σ factors regulons (Mäder et al. 2012) as assessed by SAM analysis

Regulon	Number of genes in regulon	60 min	130 min	200 min	270 min	340 min	410 min
σ^E^	171	5/0	3/1	16/8	6/2	2/2	0/10
σ^F^	62	7/0	4/2	5/2	4/3	1/1	0/1
σ^G^	108	7/0	6/0	7/4	3/1	1/0	0/0
σ^K^	103	0/0	2/0	3/2	0/2	1/1	0/3
σ^B^	159	54/0	42/0	84/5	46/0	13/0	1/2
σ^D^	81	0/0	0/0	23/0	12/1	6/0	43/0

Numbers to the left of slash sign, upregulated genes; numbers to the right of slash sign, downregulated genes

### Expression of σ^D^ regulons in Δ*prpE* mutant strain

**Fig. 4:**
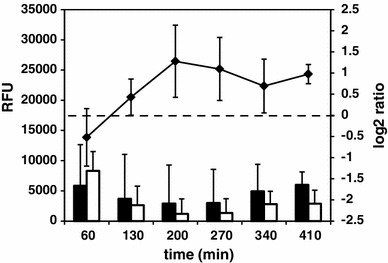
Average gene expression and Δ*prpE*/168 gene expression ratios of σ^D^ regulon. *Closed columns* gene expression in 168 wild-type strain, *open columns* gene expression in Δ*prpE* mutant strain. *Line graph* Δ*prpE*/168 gene expression ratios. *Left vertical axis values* gene expression shown as relative fluorescence units, *right vertical axis values* normalized log_2_ of ratios, *horizontal axis values* time points upon induction of sporulation. *Error bars* indicate standard deviation of gene expression or gene expression ratios

**Fig. 5:**
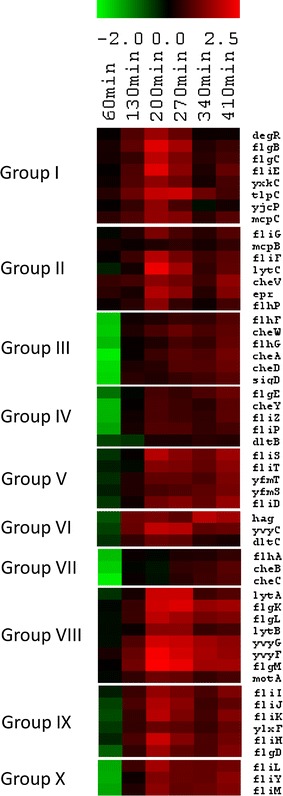
*K*-means clustering of Δ*prpE*/168 gene expression ratios of σ^D^ regulon. *Rows* represent time points from 60 to 410 min. *Red and green* indicate genes that are induced and repressed, respectively

Functional analysis revealed overrepresentation of upregulated genes of the category 4.1, Exponential and early post-exponential lifestyles, at most of time points in case of Δ*prpE* mutant strain (Fig. 2a). Genes belonging to this category are controlled mainly by σ^D^ and are responsible for mobility and chemotaxis. Therefore, we observed the overrepresentation of upregulated genes of this sigma factor regulon in Δ*prpE* strain as compared to the wild-type strain (Fig. 2c). The gene expression ratios for genes of σ^D^ regulon represented in one graph along with gene expression data (Fig. 4) show that although transcription of this regulon in both strains follows the same pattern, its overall level in Δ*prpE* strain is higher. The individual gene expression ratios of Δ*prpE*/wild-type strains were subjected to *K*-means clustering. As a result we obtained ten groups of genes with similar expression ratio pattern (Fig. 5; Figure S3 and Table S1). The expression of genes in groups I (*degR*, *flgB*, *flgC*, *fliE*, *yxkC*, *tlpC*, *yjcP*, *mcpC*), II (*fliG*, *mcpB*, *fliF*, *lytC*, *cheV*, *epr*, *flhP*), and VIII (*lytC*, *flgK*, *flgL*, *lytB*, *yvyG*, *yvyF*, *flgM*, *motA*) is higher in Δ*prpE* strain at all time points tested with the peak at 200-min time point. In case of groups III (*flhF*, *cheW*, *flhG*, *cheA*, *cheD*, *sigD*), IV (*flgE*, *cheY*, *fliZ*, *fliP*, *dltB*) and X (*fliL*, *fliY*, *fliM*) the expression of genes in mutant strain is more than twofold lower than in wild-type strain only at 60-min time point and then turns to be higher across the rest of time points. Groups V (*fliS*, *fliT*, *yfmT*, *yfmS*, *fliD*), VI (*hag*, *yvyC*, *dltC*) and IX (*fliI*, *fliJ*, *fliK*, *ylxF*, *fliH*, *flqD*) show only slight decrease of gene expression in Δ*prpE* strain as compared to the wild type at 60-min time point which then starts to be constantly higher. An individual gene expression ratio pattern is shown by genes of group VII (*flhA*, *cheB*, *cheC*) which starts with about threefold decrease at 60-min time point in mutant strain, comparable expression at 130 min in both strains and slight increase in Δ*prpE* strain at later time points.

**Fig. 6:**
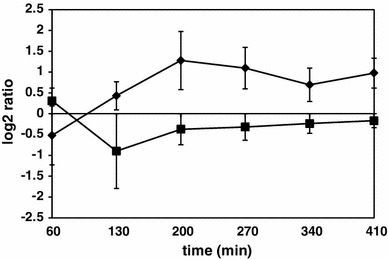
Average Δ*prpE*/168 gene expression ratios of σ^D^ and SinR regulons. *Squares* σ^D^ regulon, *diamonds* SinR regulon. *Vertical axis values* indicate normalized log_2_ of ratios, *horizontal axis values* time points upon induction of sporulation. *Error bars* indicate standard deviation of gene expression ratios

Transcription of genes important for motility as well as extracellular matrix production is regulated by Spo0A. High levels of phosphorylated Spo0A repress *fla*/*che* motility operon, while Spo0A-P is required for extracellular matrix gene expression via the activation of the regulatory protein SinI and, as a result, the transcriptional regulator SinR (Fujita et al. 2005). We have plotted Δ*prpE*/wild-type gene expression ratios of σ^D^ and SinR regulons (Table S2) in one graph (Fig. 6). The averaged expression of SinR-dependent genes is decreased in Δ*prpE* mutant as compared to the wild-type strain with the lowest level reached at 130-min time point. Since the level of Spo0A-P is controlled indirectly by a set of Rap phosphatases (Perego and Brannigan 2001; Pottathil and Lazazzera 2003) and directly by at least three other phosphatases: Spo0E (Perego and Hoch 1991; Ohlsen et al. 1994), YisI and YnzD (Perego 2001) it was interesting to compare the expression of genes coding these enzymes in wild-type and Δ*prpE* strains. Results presented in Table 3 show Δ*prpE*/wild-type strain expression ratios of these genes. While *yisI* and *ynzD* are upregulated in Δ*prpE* strain as compared to the wild type with statistically significant difference only at 200-min time point, the *rapH* gene is statistically significantly upregulated from 60 up to 270 min upon induction of sporulation.

**Table 3:** Average log_2_ Δ*prpE*/168 gene expression ratios of genes encoding Spo0A-P phosphatases and Rap phosphatases

Gene	60 min	130 min	200 min	270 min	340 min	410 min
*spo0E*	0.36	0.62	0.54	0.59	0.29	0.14
*yisI*	0.95	1.33	1.22*	0.81	0.28	0.25
*ynzD*	0.08	0.19	0.40*	0.24	0.14	0.03
*rapA*	−0.11	0.27	0.27	0.23	0.73	0.42*
*rapB*	0.29	0.11	0.31	0.05	−0.11	−0.05
*rapC*	0.31	−0.31	0.15	0.14	0.18	0.64
*rapD*	−0.06	−0.15	−0.27	−0.06	0.00	−0.23
*rapE*	1.04	0.47	0.43	0.39	0.03	0.25
*rapF*	−0.26	−0.40	0.50	0.44	0.53	0.30
*rapG*	0.01	−0.31	0.36	−0.17	−0.28	0.10
*rapH*	0.74*	0.38*	1.86*	1.61*	1.42	1.02
*rapI*	0.29	0.38	0.81	0.50	0.06	0.04
*rapJ*	0.32	0.34	−0.40	−0.26	−0.06	−0.09
*rapK*	0.27	0.47	1.53*	1.19	0.25	0.26

The numbers marked with asterisk results statistically significant as assessed by SAM analysis

### Expression of genes involved in genetic competence in Δ*prpE* mutant strain

**Fig. 7:**
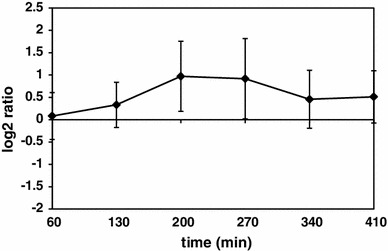
Average Δ*prpE*/168 gene expression ratios of genes involved in the development of the genetic competence. *Vertical axis values* indicate normalized log_2_ of ratios, *horizontal axis values* time points upon induction of sporulation. *Error bars* indicate standard deviation of gene expression ratios

Another functional category, which revealed upregulation of genes in Δ*prpE* mutant strain in comparison to the wild type and is controlled by Spo0A (Grossman 1995), is the category 4.1, Lifestyles—genetic competence (Fig. 2a). The genes of this functional category show overall upregulation of expression in Δ*prpE* mutant strain compared to the wild-type strain with the highest peak at 200-min time point (Fig. 7). Hierarchical clustering revealed that *comEA*-*comEC*, *comFA*-*comFC* and *comGA*-*comGG* operons are in the cluster with similar expression ratio pattern (Figure S4 and Table S3). These operons are under control of ComK competence transcription factor. The expression of *comK* gene is also upregulated in Δ*prpE* mutant cells compared to wild-type strain, with the highest peak at 270 min time point.

### Elevated expression of σ^B^ regulon in Δ*prpE* mutant strain is medium dependent

**Fig. 8:**
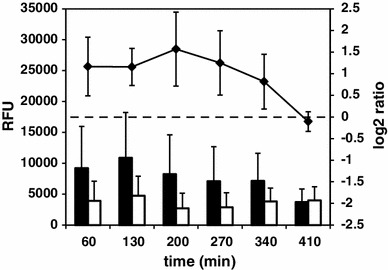
Average gene expression and Δ*prpE*/168 gene expression ratios of σ^B^ regulon. *Closed columns* gene expression in 168 wild-type strain, *open columns* gene expression in Δ*prpE* mutant strain. *Line graph* Δ*prpE*/168 gene expression ratios. *Left vertical axis values* gene expression shown as relative fluorescence units, *right vertical axis values* normalized log_2_ of ratios, *horizontal axis values* time points upon induction of sporulation. *Error bars* indicate standard deviation of gene expression or gene expression ratios

**Fig. 9:**
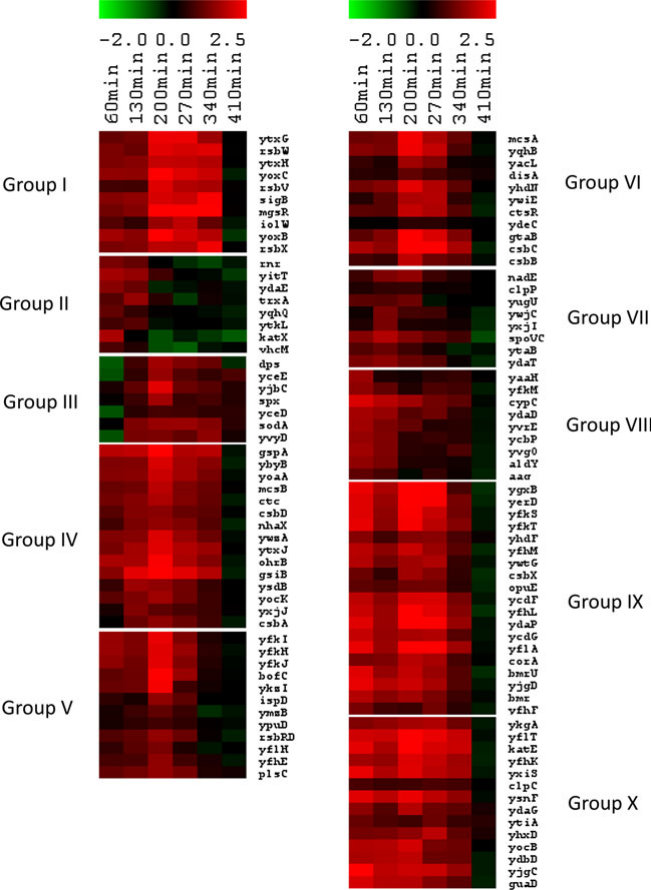
*K*-means clustering of Δ*prpE*/168 gene expression ratios of σ^B^ regulon. *Rows* represent time points from 60 to 410 min. *Red and green* indicate genes that are induced and repressed, respectively

Following the functional analysis, we have found that the category 4.3, Coping with stress, is overrepresented at all time points tested (Fig. 2a). Consequently, the same observation was made for genes belonging to the regulon of the general stress response factor σ^B^(Fig. 2c). The centroid graph of expression ratios of genes controlled by this sigma factor (159 genes) revealed overall upregulation of genes in Δ*prpE* mutant compared to the wild-type strain at all of the time points but 410 min (Fig. 8). To further analyze the expression of genes of this regulon, we performed *K*-means clustering obtaining ten groups with similar expression ratio patterns (Fig. 9; Figure S5 and Table S4). The group II (*rnr*, *yitT*, *ydaE*, *trxA*, *yqhQ*, *ytkL*, *katX*, *vhcM*) showed increased gene expression in Δ*prpE* mutant compared to the wild-type strain only at 60 and 130-min time points. At later time points gene expression was comparable in both strains. The expression of genes in the group III (*dps*, *yceE*, *yjbC*, *spx*, *yceD*, *sodA*, *yvyD*) was comparable in both strains at 60-min time point, then it started to be elevated in the mutant strain with the peak at 200-min time point to return to close to the similar level at 410-min time point. The remaining groups show the same gene expression ratio patterns of upregulation at all the time points apart from the last one, 410 min. The *sigB* gene by itself also shows clear upregulation in Δ*prpE* mutant strain with the similar pattern.

**Fig. 10:**
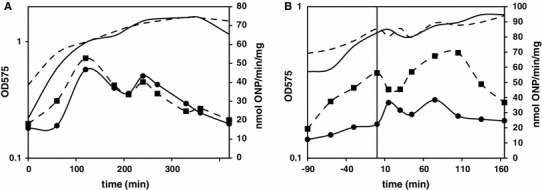
Activity of *ctc* gene promoter as assessed by β-galactosidase assay. **a** Rich medium, *horizontal axis values* indicate time upon refreshing overnight cultures. **b** Sterlini–Mandelstam and sporulation media, *horizontal axis values* indicate time upon refreshing overnight cultures relative to the resuspension of bacteria in sporulation medium indicated as 0 min. *Solid line* growth curve of strain PB198 (168 *amyE::ctc*-*lacZ*), *dashed line* growth curve of strain BAW01 (Δ*prpE amyE::ctc*-*lacZ*), *solid line with circles* β-galactosidase activity in strain PB198, *dashed line with squares* β-galactosidase activity in strain BAW01. *Left vertical axis values* indicate optical density of bacterial cultures measured at 575 nm, *right vertical axis value* β-galactosidase activity expressed in nanomoles of *o*-nitrophenol produced per minute per mg of total protein. The results are the representatives of three independent experiments

We wanted to verify whether observed upregulation of σ^B^ regulon in Δ*prpE* mutant strain was specific for sporulation process only. To do that we used the bacteria of both the wild-type and Δ*prpE* strains harboring the promoter of known σ^B^-dependent gene *ctc* fused transcriptionally with the reporter gene *lacZ* in non-essential locus *amyE*. These bacteria were grown in rich medium and the media used for the induction of sporulation and the activity of σ^B^-dependent promoter was assessed by β-galactosidase activity assay. As indicated in the Fig. 10a, the activity of *ctc* promoter follows the same pattern in both strains grown in reach medium with constant increase as the cultures are getting closer to the end of exponential growth phase and drop upon crossing the transition point. In case of media used for the induction of sporulation (Fig. 10b), the activity of *ctc* promoter follows similar pattern; although in Δ*prpE* mutant strain it is consequently higher across all time points tested.

## Discussion

The process of spore formation is controlled by a multistep genetic program involving activity of four different sigma factors of RNA polymerase as well as other transcription factors. The microarray technique has widely been used to monitor global gene expression during this developmental process (e.g. Fawcett et al. 2000; Ohashi et al. 2003; Schmalisch et al. 2010; Steil et al. 2005). In spite of obvious advantages of this technique, it is important to be aware that heterogeneity of *B. subtilis* sporulation process can be a source of a bias or even incorrect conclusions drawn from the experiments conducted with improperly synchronized bacterial cultures. In our experiments, we have used a method of inducing the sporulation widely approved as producing cultures of *B. subtilis* bacteria synchronically starting spore formation process. In addition, we have verified that both, wild-type and Δ*prpE* mutant strains initiate sporulation at the same time and with the same efficiency (Fig. 1). The *spoIIQ* gene, which promoter we have used to express GFP protein in analyzed strains, is controlled by σ^F^ subunit and its product is required for spore engulfment (Londoño-Vallejo et al. 1997) and thus it seems to be suitable for monitoring the proceeding of the sporulation process.

The PrpE protein phosphatase is thought to localize to the forespore compartment (Hinc et al. 2006), hence the deletion of the gene encoding this protein could potentially lead to altered expression of genes within the forespore. Although this hypothesis seems to be attractive, the results of transcriptional profiling of Δ*prpE* mutant strain indicate that the expression levels of genes belonging to regulons of sporulation σ factors are similar to the wild-type strain (Figure S1). Due to the fact that Δ*prpE*-produced spores are unable to initiate germination in response to l-alanine (Hinc et al. 2006), we additionally focused on the expression of germination receptors genes. The GerA receptor is thought to be responsible for induction of germination in response to l-alanine (Cabrera-Martinez et al. 2003), while GerB and GerK receptors were shown to respond to stimulation with a mixture of asparagine, glucose, fructose and potassium ions (Paidhungat and Setlow 2000). The expression of *gerAA*, *gerAB* and *gerAC* genes is unchanged in Δ*prpE* mutant strain, while *gerBA*, *gerBB* and *gerBC* are statistically significantly upregulated in the mutant strain only at two earliest time points. These time points are very early for σ^G^-dependent genes, as σ^G^ regulon is expressed no earlier than 150 min upon induction of sporulation (Steil et al. 2005). Although the expression of GerK encoding genes seems to be lowered in Δ*prpE* mutant as compared to the wild-type strain, observed differences are not statistically significant for whole operon and should not be taken into account. This observation partially contradicts our previously published data indicating that the expression of GerA receptor operon is lower in the Δ*prpE* strain as compared to the wild-type strain (Hinc et al. 2006). This discrepancy most probably results from different methodology used, because in the previous work the expression of GerA operon was analyzed using indirect method of β-galactosidase activity measurement performed with a strain harboring *lacZ* reporter gene inserted into the *gerAB*-*gerAC* region. Moreover, according to recent literature data, it is suggested that the expression level of germination receptors determines the average rate but not the heterogeneity of spore germination (Zhang et al. 2013). Hence, no germination of Δ*prpE* mutant spores in response to l-alanine does not have to correlate with lowered expression of appropriate germination receptor/receptors.

An interesting observation has been made for genes belonging to the regulon of σ^D^. The expression of σ^D^-dependent genes follows the same pattern in both Δ*prpE* and wild-type strains, but its level in Δ*prpE* mutant is elevated in comparison to the wild-type strain at 200 min and following time points (Fig. 4). The σ^D^ factor of *B. subtilis* is responsible for transcription of genes involved in motility and chemotaxis (Helmann et al. 1988; Márquez et al. 1990). It has been known for a long time that motility of sporulating *B. subtilis* decreases significantly (Nishihara and Freese 1975) and is reflected in rapid drop of σ^D^ activity upon initiation of sporulation process (Mirel and Chamberlin 1989). On the other hand, it is also known that motility, chemotaxis, development of competence and DNA uptake are alternative survival strategies of *B. subtilis* subjected to stress (Hamoen et al. 2003; Stragier 2006; Veening et al. 2008). The different cell fates are regulated by signaling network that relies primarily on the activity of three major transcriptional regulators: Spo0A, DegU and ComK (for review see Lopez et al. 2008).

A high level of DegU phosphorylation is required for the induction of exoprotease expression. In contrast, high levels of DegU-P repress motility as this protein directly binds the regulatory region of the *fla*/*che* motility and chemotaxis operon (Tsukahara and Ogura 2008). In our experiments, we have not observed differences in the expression of genes encoding extracellular degradative enzymes between Δ*prpE* and the wild-type strains (data not shown). This leads to the conclusion that DegU probably does not directly contribute to elevated expression of σ^D^ regulon in *prpE*-deleted strain.

In the sessile state *B. subtilis* cells, especially those that form biofilms are able to produce extracellular matrix. Its production requires activation of two operons: *epsA*-*O*, responsible for production of the exopolysaccharide component and the *yqxM*-*sipW*-*tasA* operon, responsible for the production and secretion of the major protein component of the matrix (Branda et al. 2004; Kearns et al. 2005). These operons are controlled by an inhibitor, SinR. The activity of SinR is negatively regulated by a regulatory protein SinI, which is expressed only in cells in which Spo0A has been activated (Chai et al. 2008). On the other hand, high levels of Spo0A-P repress the *fla*/*che* operon (Fujita et al. 2005). Thus, the level of phosphorylated form of Spo0A in the cell can lead to a switch between motility and extracellular matrix production. The expression of SinR regulon is lowered in Δ*prpE* mutant as compared to the wild-type strain (Fig. 6), which can correlate with elevated expression of σ^D^ regulon.

The elevated expression in Δ*prpE* mutant as compared to the wild-type strain was also observed for genes involved in genetic competence (Fig. 7). The genetic competence development is governed by the master regulator ComK, which controls the initiation of the cascade of the genes involved in this process (Dubnau 1991; van Sinderen et al. 1995). The activation and accumulation of ComK is controlled by products of several genes including *abrB*, *sinR* and *degU* (Hahn et al. 1994; van Sinderen and Venema 1994). The control via AbrB is influenced by Spo0A, since the transcription of *abrB* is repressed by Spo0A-P (78,104). According to the work of Mirouze and co-workers, Spo0A-P directly binds to the promoter region of *comK* and activates or represses its activity dependently on the concentration in the cell (Mirouze et al. 2012). Therefore, the upregulation of genes involved in development of genetic competence, especially ComK-dependent genes (Figure S3) observed in Δ*prpE* strain may be explained by the regulatory action of Spo0A.

The results of our analysis point to the activity of Spo0A regulator as a probable cause of changed expression of above described groups of genes. The efficiency of spore formation as well as the timing of sporulation process seems to be unaffected in Δ*prpE* strain (Hinc et al. 2006 and Fig. 1), hence any potential changes in the level of Spo0A-P in mutant cells should be rather discrete and most probably occur upon successful initiation of sporulation. The expression of *spo0A* gene is similar in both the strains Δ*prpE* mutant and the wild type (data not shown). Our attention was drawn by phosphatases of Spo0A-P, which are responsible for inactivation of this protein. The genes encoding two direct phosphatases of Spo0A-P, *spo0E* and *yisI*, and one gene coding for Rap phosphatase, *rapH*, involved in regulation of phosphorelay (Parashar et al. 2011) are upregulated in Δ*prpE* strain. The combined action of these proteins may lead to more rapid drop of Spo0A-P level in Δ*prpE* strain resulting in partial de-repression of σ^D^ regulon and the genes involved in the development of genetic competence. As another effect of this drop, the repression of SinR regulon might occur via activation of SinR antagonist, SinI.

An unexpected observation has been made for genes belonging to the regulon of σ^B^, the key regulator of the general stress response in *B. subtilis*. Most of σ^B^-dependent genes are upregulated in Δ*prpE* mutant strain (Fig. 8), which clearly suggests higher level of stress response in sporulating mutant bacteria. One could suggest that the shift of growing cultures from rich growth medium to very minimal sporulation medium, which takes place at the induction of sporulation procedure, may lead to a starvation and thus induction of stress response. We have verified this assumption by analyzing the expression of *ctc*, a well-known general stress response genes, in bacteria growing in rich medium or media used for induction of sporulation. While the activity of *ctc* promoter in rich medium did not differ in both strains (Fig. 10a), in Sterlini–Mandelstam (minimal) medium used for growing bacteria as well as very minimal resuspension medium used for induction of sporulation, the activity of *p*_*ctc*_ was elevated in Δ*prpE* mutant strain in the samples from each time point analyzed (Fig. 10b). This observation leads to the conclusions that elevated level of general stress response in the mutant strain is not sporulation specific. Moreover, it is connected with the type of media used for cultivation of bacteria. It seems plausible that the deletion of *prpE* gene leads to impaired utilization of amino acids as carbon source, nevertheless further investigation is needed for the verification of such hypothesis.

Taken together our results point to PrpE phosphatase as a protein involved in the regulation of processes described above via yet undefined mechanism, most probably influencing the activity of Spo0A regulator in sporulating *B. subtilis* cells. This hypothesis is an addition to former observations that the presence of this phosphatase seems to be important for establishing proper resistance of spores as well as for germination. Such conclusions place PrpE protein in the center of interest for research conducted on basic cellular and developmental processes of *B. subtilis*.

## Electronic supplementary material

Below is the link to the electronic supplementary material.

Supplementary material 1 (PDF 1,292 kb)
