# Supplementary material for: A genome-wide transcriptional profiling of sporulating Bacillus subtilis strain lacking PrpE protein phosphatase
Source: Mol Genet Genomics. 2013 Jul 4;288(10):469–81. doi: 10.1007/s00438-013-0763-7 (PMC3782651; doi:10.1007/s00438-013-0763-7)
Supplement: Supplementary file 1 — Supplementary material 1 (PDF 1,292 kb) [file 438_2013_763_MOESM1_ESM.pdf]

A genome-wide transcriptional profiling of sporulating *Bacillus subtilis* strain lacking PrpE protein phosphatase

Molecular Genetics and Genomics - supplementary material

Adam Iwanicki[1], Krzysztof Hinc[1], Anna Ronowicz[2], Arkadiusz Piotrowski[2], Aleksandra Wołoszyk[1], Michał Obuchowski[1*]

[1]Laboratory of Molecular Bacteriology

Intercollegiate Faculty of Biotechnology University of Gdańsk and Medical University of Gdańsk

Dębinki 1, 80-211 Gdańsk, Poland

[2]Department of Biology and Pharmaceutical Botany, Medical University of Gdansk, Hallera 107, 80-416 Gdansk, Poland

*Corresponding author

Contact details:

Michał Obuchowski

Laboratory of Molecular Bacteriology

Intercollegiate Faculty of Biotechnology University of Gdańsk and Medical University of Gdańsk

Dębinki 1, 80-211 Gdańsk, Poland

Tel. +48 58 3491484

Fax. +48 58 3491445

Email address: obuchowk@biotech.ug.gda.pl

Table S1.

K-means clustered regulon of σ^D. Numbers in the table indicate $\log_2$ of Δ*prpE*/168 gene expression ratios.

Gene	60min	130min	200min	270min	340min	410min	Group
degR	0.06	0.79	1.52	0.99	0.12	0.13	I
flgB	0.24	0.94	2.20	1.62	0.33	0.57	
flgC	0.21	0.54	1.85	1.23	0.43	0.69	
fliE	0.06	0.76	2.22	1.36	0.30	0.97	
yxkC	0.13	0.79	1.40	1.04	0.21	0.71	
tlpC	0.37	1.04	1.92	2.03	1.05	0.68	
yjcP	0.06	0.52	1.40	0.72	-0.11	0.21	
mcpC	0.44	0.77	1.44	1.30	0.39	0.84	
fliG	-0.05	0.35	1.22	0.72	0.40	0.81	II
mcpB	0.24	0.12	0.32	0.46	0.23	0.50	
fliF	0.05	0.60	1.79	1.14	0.60	0.88	
lytC	-0.24	0.19	2.35	1.59	0.50	0.93	
cheV	0.53	0.68	1.37	1.10	0.61	1.42	
epr	0.43	0.24	1.73	1.36	0.38	1.28	
flhP	0.12	0.40	1.36	0.50	0.15	0.67	
flhF	-1.54	0.18	0.33	0.55	0.49	0.80	III
cheW	-1.60	0.31	0.71	1.00	0.72	0.92	
flhG	-1.47	0.08	0.31	0.69	0.53	0.98	
cheA	-1.86	0.08	0.59	0.86	0.75	1.13	
cheD	-1.72	0.29	0.40	0.81	0.80	1.04	
sigD	-1.68	0.42	0.32	0.53	0.64	0.86	
flgE	-0.89	-0.04	0.69	0.53	0.75	1.04	IV
cheY	-1.43	0.06	0.75	0.61	0.41	0.87	
fliZ	-1.40	0.16	0.74	0.61	0.49	0.88	
fliP	-1.30	0.24	0.79	0.77	0.50	0.68	
dltB	-0.52	-0.39	0.28	0.28	0.43	0.36	
fliS	-0.38	0.06	1.78	1.34	1.02	1.55	V
fliT	-0.22	-0.07	1.30	1.21	0.96	1.37	
yfmT	-0.34	0.33	0.95	0.94	0.74	1.22	
yfmS	-0.23	0.32	0.70	0.78	0.92	1.16	
fliD	-0.42	0.17	1.08	1.28	0.95	1.46	
hag	-0.66	1.07	1.18	0.89	1.83	1.58	VI
yvyC	-0.50	1.02	1.91	1.81	0.92	1.02	
dltC	-0.38	0.51	1.00	0.90	0.36	0.25	
flhA	-1.49	0.12	-0.05	0.30	0.32	0.51	VII
cheB	-1.63	-0.04	-0.15	0.49	0.57	0.86	
cheC	-1.88	0.10	-0.12	0.40	0.62	0.79	
lytA	-0.38	0.24	2.00	2.00	0.60	0.81	VIII
flgK	-0.05	0.55	2.05	2.18	1.48	1.59	
flgL	-0.09	0.57	1.84	1.57	1.15	1.25	
lytB	-0.08	0.12	1.35	1.36	0.54	0.70	
yvyG	-0.09	0.78	2.07	1.97	1.57	1.40	
yvyF	0.26	1.01	2.41	2.08	1.65	1.45	
flgM	0.03	1.11	2.84	2.32	1.58	1.61	
motA	-0.05	0.48	1.07	1.00	0.52	0.70	
fliI	-0.22	0.59	1.45	1.16	0.72	1.24	IX
fliJ	-0.52	0.73	1.71	1.35	0.62	0.93	

fliK	-0.59	0.53	1.81	1.22	0.68	1.29	
ylxF	-0.45	0.51	1.02	0.89	0.57	0.99	
fliH	-0.27	0.74	1.99	1.31	0.75	1.11	
flgD	-0.75	0.49	1.54	0.92	0.74	1.26	
fliL	-1.32	0.61	1.60	1.03	0.97	1.12	X
fliY	-1.36	0.14	1.26	1.02	0.98	1.35	
fliM	-1.30	0.33	1.36	0.96	1.05	1.27	

Table S2.

K-means clustered regulon of SinR. Numbers in the table indicate log_2 of Δ*prpE*/168 gene expression ratios.

Gene	60min	130min	200min	270min	340min	410min
spoVG	-0.09	-0.40	-0.07	-0.51	-0.38	0.02
aprE	0.66	0.47	1.53	0.86	-0.40	-0.28
rok	0.43	0.14	1.15	0.32	-0.30	0.22
spoIIGA	0.63	-0.71	0.16	-0.25	-0.26	-0.09
sigE	0.63	-0.67	1.21	0.64	-0.45	0.05
sigG	0.76	-0.86	-0.41	-0.44	-0.50	-0.21
sigF	0.27	-0.43	-0.47	-0.72	-0.14	-0.40
spoIIAB	0.31	-0.43	-0.13	-0.16	-0.34	-0.33
spoIIAA	0.24	-0.23	-0.29	-0.38	-0.09	-0.32
tasA	0.54	-1.51	-1.25	-1.02	-0.56	-0.25
sipW	0.42	-1.17	-1.13	-0.84	-0.44	-0.33
tapA	0.36	-0.94	-1.31	-0.81	-0.01	-0.12
yvgN	-1.71	0.17	0.48	0.08	-0.07	0.14
lutC	1.06	-0.55	-0.03	-0.22	-0.23	0.11
lutB	1.25	-0.50	0.18	0.04	-0.14	0.28
lutA	1.57	-0.69	0.05	0.21	-0.24	0.57
epsO	0.00	-1.10	-0.01	-0.08	-0.11	-0.26
epsN	-0.08	-1.44	-0.90	-0.23	-0.36	-0.48
epsM	-0.02	-1.29	-0.77	-0.20	-0.11	-0.38
epsL	-0.11	-1.46	-0.62	-0.31	-0.36	-0.41
epsK	0.00	-1.69	-1.28	-0.64	-0.35	-0.39
epsJ	0.10	-1.68	-1.11	-0.47	-0.07	-0.35
epsI	0.02	-1.58	-1.07	-0.50	-0.31	-0.39
epsH	0.08	-1.65	-1.41	-0.58	-0.12	-0.35
epsG	0.12	-1.83	-1.98	-1.18	-0.30	-0.51
epsF	0.12	-1.50	-1.02	-0.53	-0.23	-0.34
epsE	0.07	-1.44	-0.13	-0.49	-0.10	-0.35
epsD	0.19	-1.80	-0.61	-0.71	-0.26	-0.36
epsC	0.29	-1.42	-0.79	-0.89	-0.20	-0.34
epsB	0.57	-1.43	-1.49	-0.96	-0.32	-0.59
epsA	0.65	-1.28	-0.99	-0.84	-0.30	-0.38
slrR	0.60	-0.61	-0.70	-0.43	-0.14	-0.41
ywbD	0.37	0.20	0.05	-0.10	-0.18	-0.01
epr	0.43	0.24	1.73	1.36	0.38	1.28
rapG	0.01	-0.31	0.36	-0.17	-0.28	0.10

Table S3.

K-means clustered genes belonging to the functional category 4.1. Lifestyles – genetic competence. Numbers in the table indicate $\log_2$ of Δ*prpE*/168 gene expression ratios.

Gene	60min	130min	200min	270min	340min	410min
addA	-0.20	0.03	0.22	0.45	0.17	0.10
addB	0.11	0.07	0.18	0.35	0.14	0.00
bdbC	-0.43	-0.50	1.09	0.35	-0.38	0.28
bdbD	-0.32	-0.43	0.94	0.31	0.06	0.31
coiA	0.46	0.67	0.85	1.33	0.64	0.40
comA	-0.36	-0.09	1.03	0.71	0.32	0.74
comC	0.34	-0.51	-0.49	0.38	0.17	0.04
comEA	0.90	0.74	0.98	1.48	0.28	0.14
comEB	0.61	0.89	1.28	1.49	0.28	0.11
comEC	0.28	0.20	0.28	0.55	0.08	-0.02
comER	0.69	0.22	-0.17	-0.15	-0.09	-0.08
comFA	0.76	1.36	1.88	2.00	1.58	1.43
comFB	0.50	1.47	2.78	2.20	1.79	1.68
comFC	0.52	1.02	1.90	1.90	1.88	1.41
comGA	1.18	1.02	2.01	2.28	1.57	1.62
comGB	0.83	0.90	2.06	2.34	1.52	1.47
comGC	0.87	1.02	2.34	2.81	1.55	1.58
comGD	0.72	0.85	2.01	2.56	1.47	1.52
comGE	0.64	0.80	1.91	2.34	1.30	1.45
comGF	0.45	0.55	1.61	2.40	1.19	1.16
comGG	0.15	0.27	1.49	2.42	0.81	0.89
comK	0.72	1.07	0.92	1.23	0.38	0.52
comN	-0.54	0.12	0.50	0.55	0.08	-0.02
comP	-0.10	-0.28	0.99	0.48	0.42	0.38
comQ	-0.21	-0.21	0.90	0.37	0.45	0.34
comS	0.35	0.04	0.39	0.55	0.72	0.60
comX	-0.27	0.25	2.03	1.12	0.78	0.70
comZ	-0.35	0.61	0.63	0.42	-0.19	-0.08
dprA	0.46	0.27	0.81	1.65	1.06	0.74
mecA	-0.03	1.16	0.72	0.44	0.16	-0.12
med	0.29	0.34	0.04	0.00	0.08	0.00
nin	0.34	0.99	2.15	2.08	0.93	1.13
nucA	0.15	0.89	2.05	1.88	1.20	1.34
oppA	-0.68	0.31	1.34	0.90	-0.16	0.12
oppB	-0.76	0.27	1.09	0.65	0.05	0.13
oppC	-0.82	0.26	0.98	0.63	-0.01	0.07
oppD	-0.82	-0.01	0.64	0.17	-0.13	0.18
oppF	-0.70	-0.05	0.77	0.05	-0.40	-0.08
phrC	0.19	-0.13	0.48	-0.51	0.15	0.60
phrF	-0.08	-0.12	0.27	0.31	0.90	0.51
phrK	-0.98	0.20	1.04	0.48	0.26	-0.15
pnpA	-0.04	0.01	-0.54	-0.79	-0.66	0.24
radC	-0.61	0.04	0.47	0.96	0.13	0.08
rapC	0.31	-0.31	0.15	0.14	0.18	0.64
rapD	-0.06	-0.15	-0.27	-0.06	0.00	-0.23
rapF	-0.26	-0.40	0.50	0.44	0.53	0.30

rapK	0.27	0.47	1.53	1.19	0.25	0.26
recA	0.20	0.75	1.72	1.33	1.16	1.71
rok	0.43	0.14	1.15	0.32	-0.30	0.22
sbcC	-0.49	-0.01	-0.18	-0.47	-0.69	-0.11
sbcD	-0.41	0.00	0.06	0.30	0.17	0.05
yvcJ	0.01	0.18	0.91	0.30	-0.15	0.26

Table S4.

K-means clustered regulon of σ^B. Numbers in the table indicate $\log_2$ of Δ*prpE*/168 gene expression ratios.

Gene	60min	130min	200min	270min	340min	410min	Group
ytxG	1.00	1.14	2.31	2.29	1.53	0.01	I
rsbW	1.22	1.05	2.19	2.07	2.17	-0.05	
ytxH	1.23	1.34	1.91	1.89	1.75	0.01	
yoxC	1.41	1.40	2.91	2.16	1.96	-0.26	
rsbV	0.68	0.68	2.15	1.74	1.64	0.11	
sigB	1.31	1.42	2.10	1.97	2.43	0.09	
SmgsR	1.13	1.31	3.56	3.12	2.45	0.11	
iolW	0.84	0.51	1.49	1.87	1.00	-0.19	
yoxB	1.60	1.36	2.58	2.03	1.76	-0.42	
rsbX	1.19	1.30	1.82	1.78	2.35	-0.03	
rnr	1.19	0.99	0.07	-0.36	-0.54	-0.20	II
yitT	1.60	1.45	0.66	-0.01	-0.11	-0.45	
ydaE	1.36	1.04	-0.31	-0.15	0.18	-0.16	
trxA	0.86	1.46	0.45	-0.46	0.24	-0.14	
yqhQ	1.00	0.55	0.12	0.04	-0.14	-0.27	
ytkL	0.68	0.88	-0.16	0.08	0.04	-0.17	
katX	1.68	0.00	-0.62	-0.23	-0.47	-0.72	
yhcM	0.79	0.39	-0.62	-0.74	-0.18	-0.08	
dps	-0.71	0.67	1.58	1.26	1.05	-0.35	III
yceE	-0.64	0.51	1.49	0.86	0.55	0.78	
yjbC	0.31	0.95	2.20	1.07	0.89	0.41	
spx	0.12	0.63	1.60	0.61	0.72	0.40	
yceD	-0.66	0.45	0.71	0.67	0.51	0.48	
sodA	0.01	1.24	1.49	1.53	1.31	0.46	
yvyD	-0.65	1.17	1.23	0.99	1.40	0.28	
gspA	1.80	1.86	2.67	1.73	1.80	-0.16	IV
ybyB	1.25	1.29	2.11	1.61	1.35	-0.26	
yoaA	1.31	1.35	1.96	1.58	1.18	-0.13	
mcsB	1.06	1.12	1.81	1.25	1.05	0.09	
ctc	1.14	1.31	1.69	1.28	1.01	-0.24	
csbD	0.89	1.30	1.37	1.05	0.93	-0.08	
nhaX	0.67	1.21	1.45	1.27	0.86	-0.29	
ywzA	1.28	1.34	2.14	1.49	1.06	-0.12	
ytxJ	1.18	1.60	2.19	1.74	1.53	-0.03	
ohrB	1.66	1.64	2.43	1.67	1.30	-0.18	
gsiB	1.34	2.21	3.36	2.19	1.75	-0.26	
ysdB	0.58	1.53	1.42	1.13	0.59	0.03	
yocK	0.91	1.23	1.57	1.11	0.64	0.03	
yxjJ	0.31	1.29	0.95	0.76	0.62	0.08	
csbA	0.10	1.05	1.39	1.06	0.66	-0.22	
yfkI	1.47	1.23	2.27	1.26	0.38	0.04	V
yfkH	1.50	1.25	2.18	1.51	0.47	-0.15	
yfkJ	1.40	1.13	2.19	1.30	0.41	-0.08	
bofC	0.95	1.08	2.56	1.53	0.31	-0.06	
ykzI	1.03	1.09	2.38	0.64	0.24	-0.03	
ispD	0.50	0.29	1.41	0.93	-0.04	-0.04	
ymzB	0.30	0.62	0.96	0.60	-0.36	-0.18	

ypuD	0.29	0.50	0.67	0.51	0.11	-0.11	
rsbRD	0.83	0.96	1.43	1.04	0.14	-0.27	
yflH	0.52	0.89	1.19	0.36	-0.20	-0.04	
yfhE	0.72	0.75	1.40	0.67	0.31	-0.21	
plsC	0.97	1.13	1.43	1.16	0.38	0.25	
mcsA	1.09	1.16	2.37	1.67	1.01	-0.08	VI
yqhB	1.32	1.19	2.33	1.91	0.79	-0.15	
yacL	0.58	0.35	1.57	1.27	0.21	0.16	
disA	0.47	0.41	0.98	0.71	0.42	0.25	
yhdN	1.14	1.31	2.23	1.74	0.93	0.00	
ywiE	0.41	0.87	1.71	1.76	0.51	-0.22	
ctsR	0.92	0.88	1.92	1.71	0.87	-0.30	
ydeC	0.11	0.10	0.48	0.45	0.16	0.00	
gtaB	0.91	1.29	2.62	2.11	1.25	0.04	
csbC	1.66	1.41	3.39	2.99	1.67	-0.30	
csbB	0.70	0.59	1.86	1.09	0.88	-0.12	
nadE	0.62	1.26	1.42	0.63	0.37	0.07	VII
clpP	0.37	0.55	0.56	0.20	0.21	0.03	
yugU	0.91	0.89	1.08	-0.17	0.04	-0.01	
ywjC	0.21	1.22	0.71	0.67	0.14	-0.31	
yxjI	0.37	1.19	0.34	0.32	0.26	-0.34	
spoVC	1.33	1.75	1.30	0.96	0.68	-0.59	
ytaB	0.84	1.05	0.65	0.28	-0.29	-0.14	
ydaT	1.24	1.40	1.06	0.87	0.17	-0.33	
yaaH	1.53	0.36	0.24	0.49	0.55	0.09	VIII
yfkM	1.39	0.98	0.37	0.78	0.69	-0.24	
cypC	2.13	1.80	1.59	1.15	0.94	-0.09	
ydaD	1.61	1.48	0.88	1.12	0.52	-0.19	
yvrE	1.58	1.23	1.00	0.41	0.43	-0.16	
ycbP	1.47	1.27	0.47	0.60	0.45	-0.19	
yvgO	1.57	1.26	0.47	0.40	0.79	-0.23	
aldY	1.42	1.21	0.37	0.37	0.24	-0.23	
aag	0.97	0.72	-0.10	0.56	0.20	-0.29	
ygxB	2.29	1.68	2.56	2.43	0.75	-0.39	IX
yerD	2.16	1.53	2.72	2.43	1.17	-0.31	
yfkS	2.16	1.43	2.44	1.96	1.14	-0.20	
yfkT	2.25	1.38	2.39	1.83	1.32	-0.19	
yhdF	1.34	0.69	1.30	1.36	0.76	0.18	
yfhM	1.80	1.39	1.95	1.53	0.89	-0.33	
ywtG	1.74	1.14	1.58	1.71	0.57	-0.18	
csbX	1.06	0.55	1.58	1.31	0.51	-0.35	
opuE	0.95	0.82	1.13	1.14	0.46	-0.31	
ycdF	1.85	1.40	2.11	2.12	1.19	-0.19	
yfhL	1.95	1.51	2.55	2.20	1.33	-0.39	
ydaP	2.19	1.52	2.47	2.45	1.49	-0.16	
ycdG	1.90	1.48	1.66	1.74	0.87	-0.19	
yflA	2.28	1.51	3.02	2.74	1.57	-0.12	
corA	1.20	1.25	1.81	1.64	0.72	-0.06	
bmrU	2.18	1.48	1.90	1.50	0.86	-0.36	
yjgD	2.26	1.79	1.98	1.65	0.85	-0.20	
bmr	1.77	1.36	1.63	1.22	0.63	-0.10	

yfhF	1.19	0.69	0.61	1.13	0.45	-0.25	
ykgA	1.27	1.41	1.62	1.70	1.11	-0.22	X
yflT	2.04	1.98	2.57	2.28	1.93	-0.14	
katE	2.22	1.66	2.56	2.09	1.97	-0.26	
yfhK	1.58	1.45	2.15	1.91	1.55	-0.29	
yxiS	2.28	1.72	2.18	1.82	1.62	-0.21	
clpC	0.76	0.62	0.67	0.78	0.69	-0.03	
ysnF	2.17	1.83	2.28	1.84	1.42	-0.12	
ydaG	1.38	0.86	1.77	1.11	0.95	0.31	
ytiA	1.02	0.78	0.66	1.07	0.60	0.43	
yhxD	1.15	1.21	1.41	1.82	0.95	0.33	
yocB	1.73	1.75	2.04	1.18	1.09	0.01	
ydbD	1.65	1.73	1.85	1.07	1.05	-0.23	
yjgC	2.56	1.84	1.97	1.93	1.46	-0.23	
guaD	2.09	1.76	1.76	1.31	0.91	-0.29	

Supplementary figures legends

Figure S1

MA plots of Δ*prpE*/wild-type gene expression ratios. Vertical axis values indicate normalized $\log_2$ of ratios (M), horizontal axis values – $\log_2$ average expression intensity (A). Genes differentially expressed as assessed by the SAM analysis are shown in black.

Figure S2

Averaged Δ*prpE*/168 gene expression ratios of sporulation σ factors regulons. Vertical axis values indicate normalized $\log_2$ of ratios, horizontal axis values – time points upon induction of sporulation. Error bars indicate standard deviation of gene expression ratios.

Figure S3

Δ*prpE*/168 gene expression ratios of σ^D regulon in groups produced by K-means clustering. Thin lines – gene expression ratio of individual genes. Thick line – averaged gene expression ratio in the group. Vertical axis values indicate normalized $\log_2$ of ratios. Horizontal axis values – time points upon induction of sporulation.

Figure S4

K-means clustering of Δ*prpE*/168 gene expression ratios of genes belonging to the functional category 4.1. Lifestyles - genetic competence. Rows represent time points from 60 min to 410 min. Red and green indicate genes that are induced and repressed, respectively.

Figure S5

Δ*prpE*/168 gene expression of σ^B regulon ratios in groups produced by K-means clustering. Thin lines – gene expression ratio of individual genes. Thick line – averaged gene expression ratio in the group. Vertical axis values indicate normalized $\log_2$ of ratios. Horizontal axis values – time points upon induction of sporulation.

Figure S1.

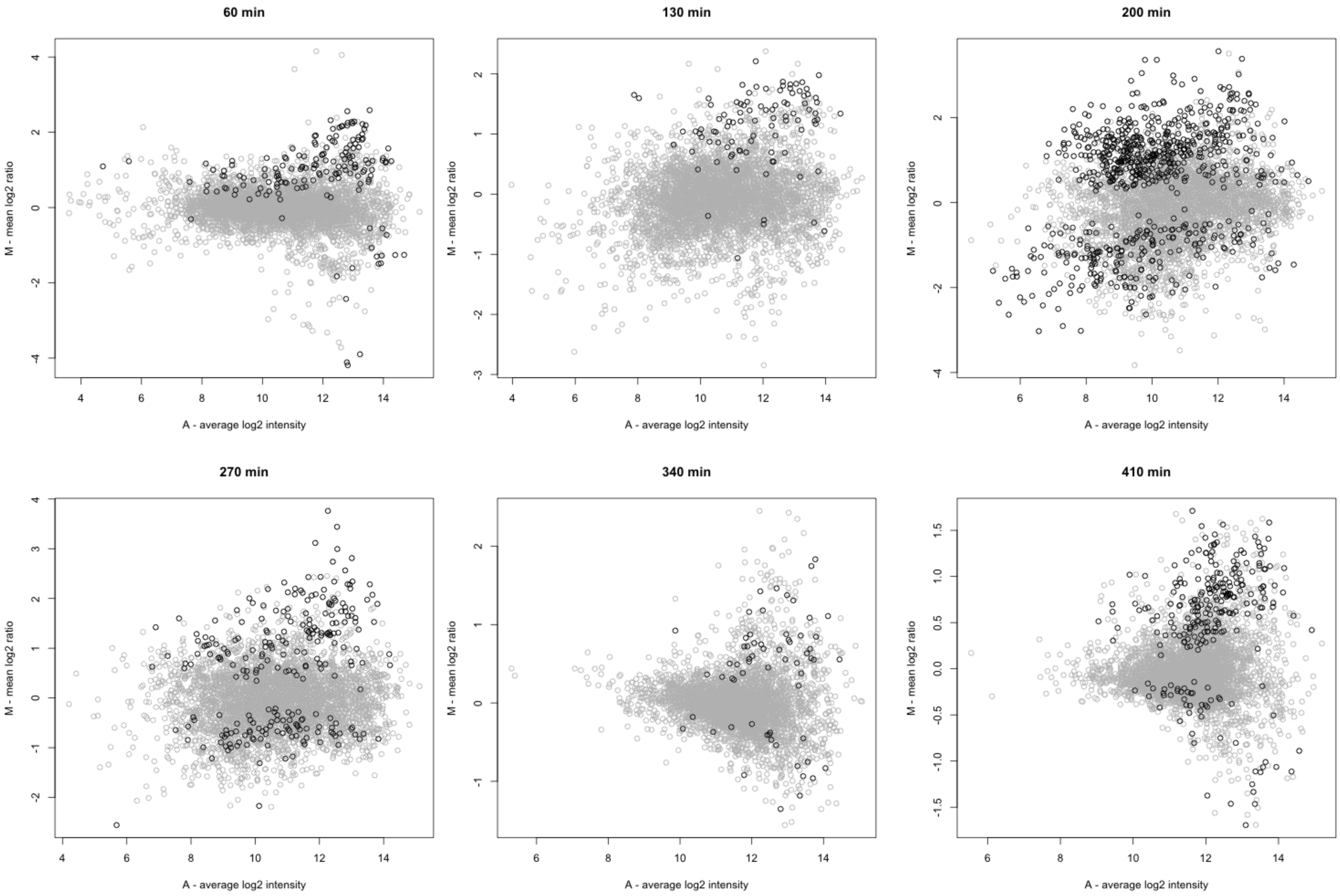
60 min
130 min
200 min
270 min
340 min
410 min
M - mean log2 ratio
A - average log2 intensity

Figure S2.

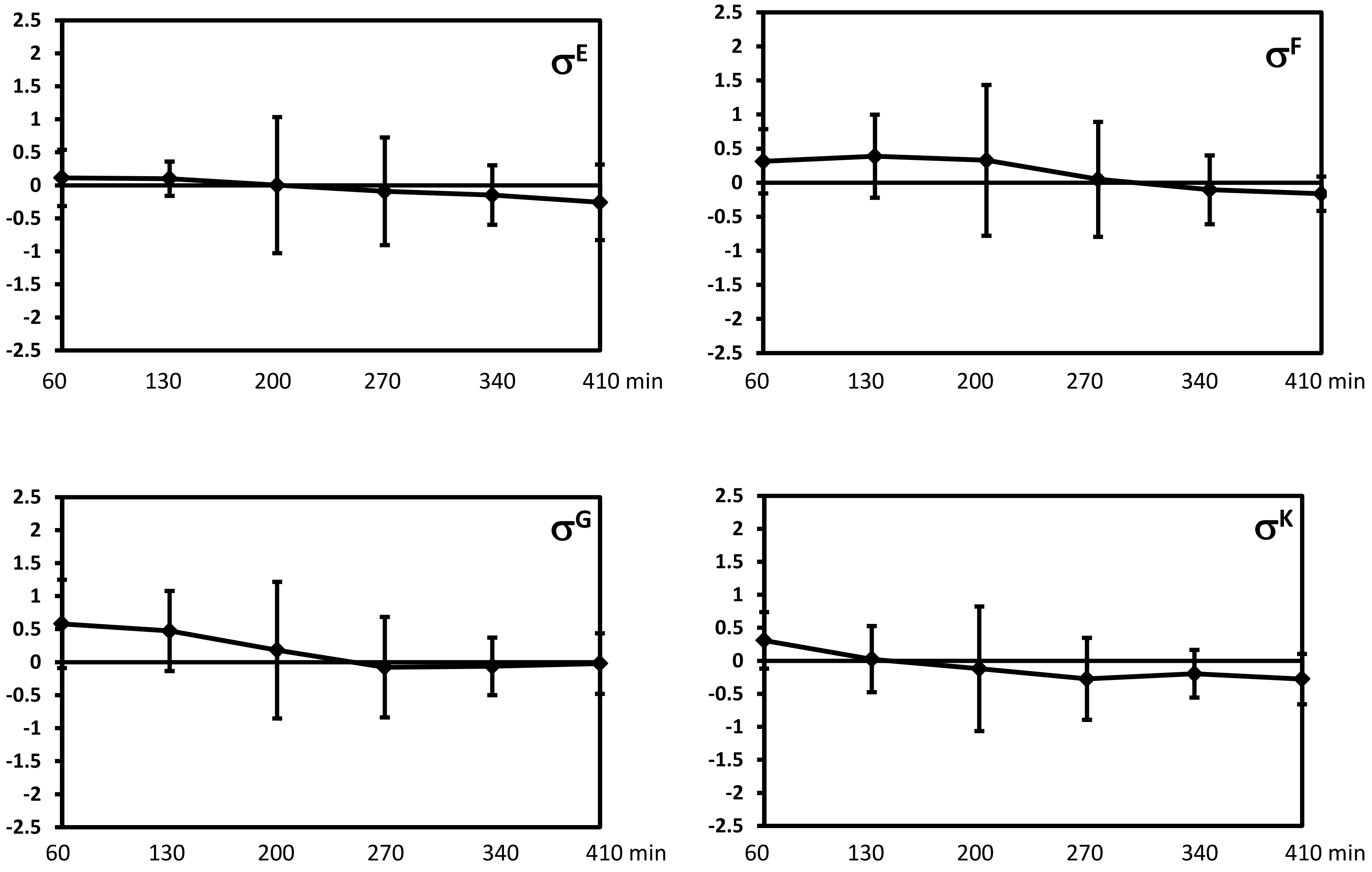

Figure S3.

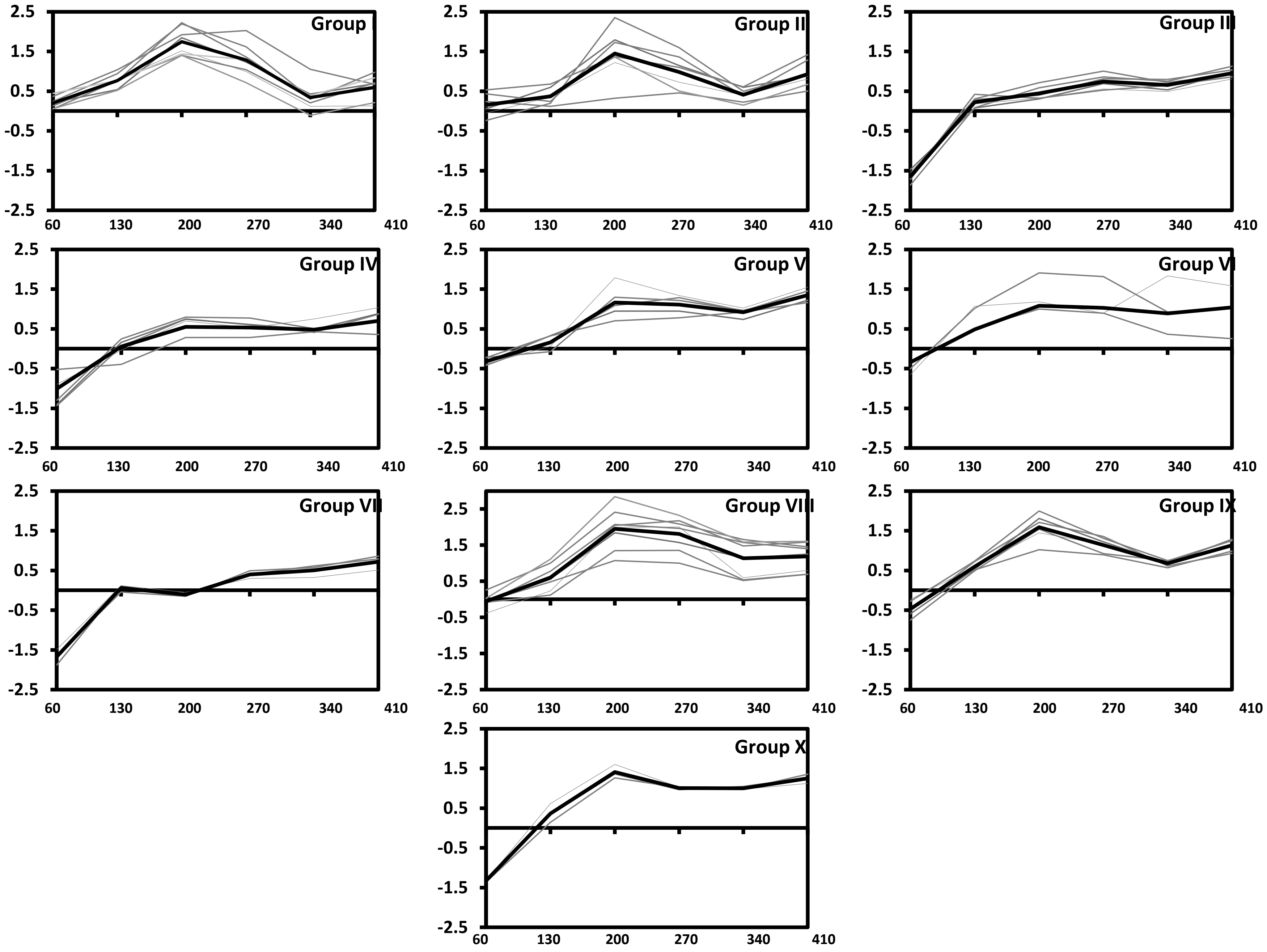

Group
Group II
Group III
Group IV
Group V
Group VI
Group VII
Group VIII
Group IX
Group X
2.5
1.5
0.5
-0.5
-1.5
-2.5
60
130
200
270
340
410

Figure S4.

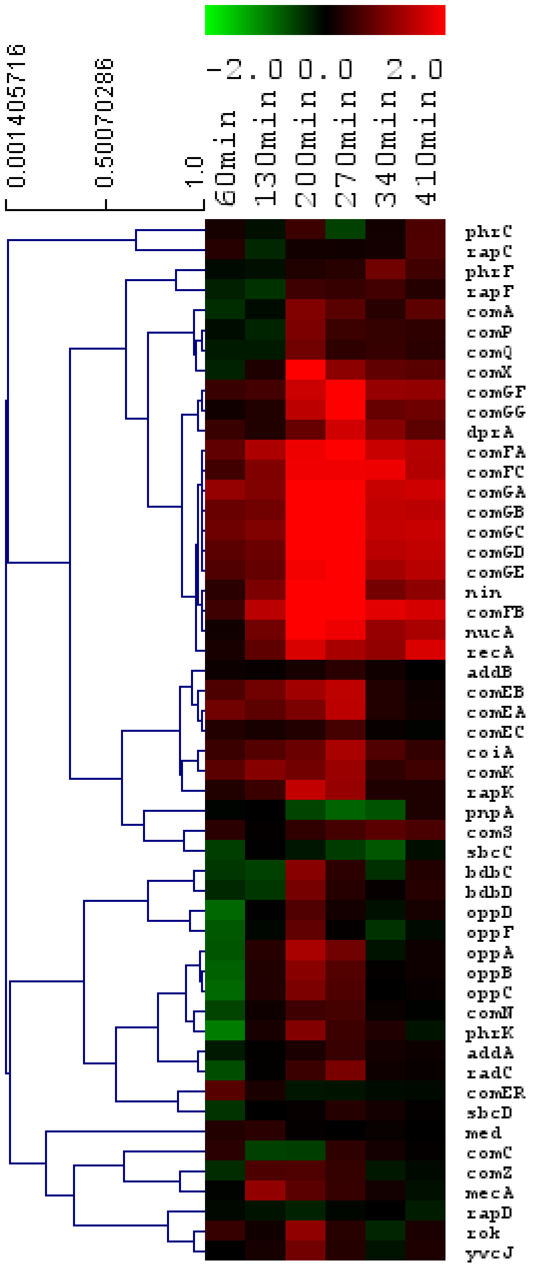
-2.0 0.0 2.0
0.001405716
0.50070286
1.0
60min
130min
200min
270min
340min
410min
phrC
rapC
phrF
rapF
comA
comP
comQ
comX
comGF
comGG
dprA
comFA
comFC
comGA
comGB
comGC
comGD
comGE
nin
comFB
nucA
recA
addB
comEB
comEA
comEC
coiA
comK
rapK
pnpA
comS
sbcC
bdbC
bdbD
oppD
oppF
oppA
oppB
oppC
comN
phrK
addA
radC
comER
sbcD
med
comC
comZ
mecA
rapD
rok
ywcJ

Figure S5

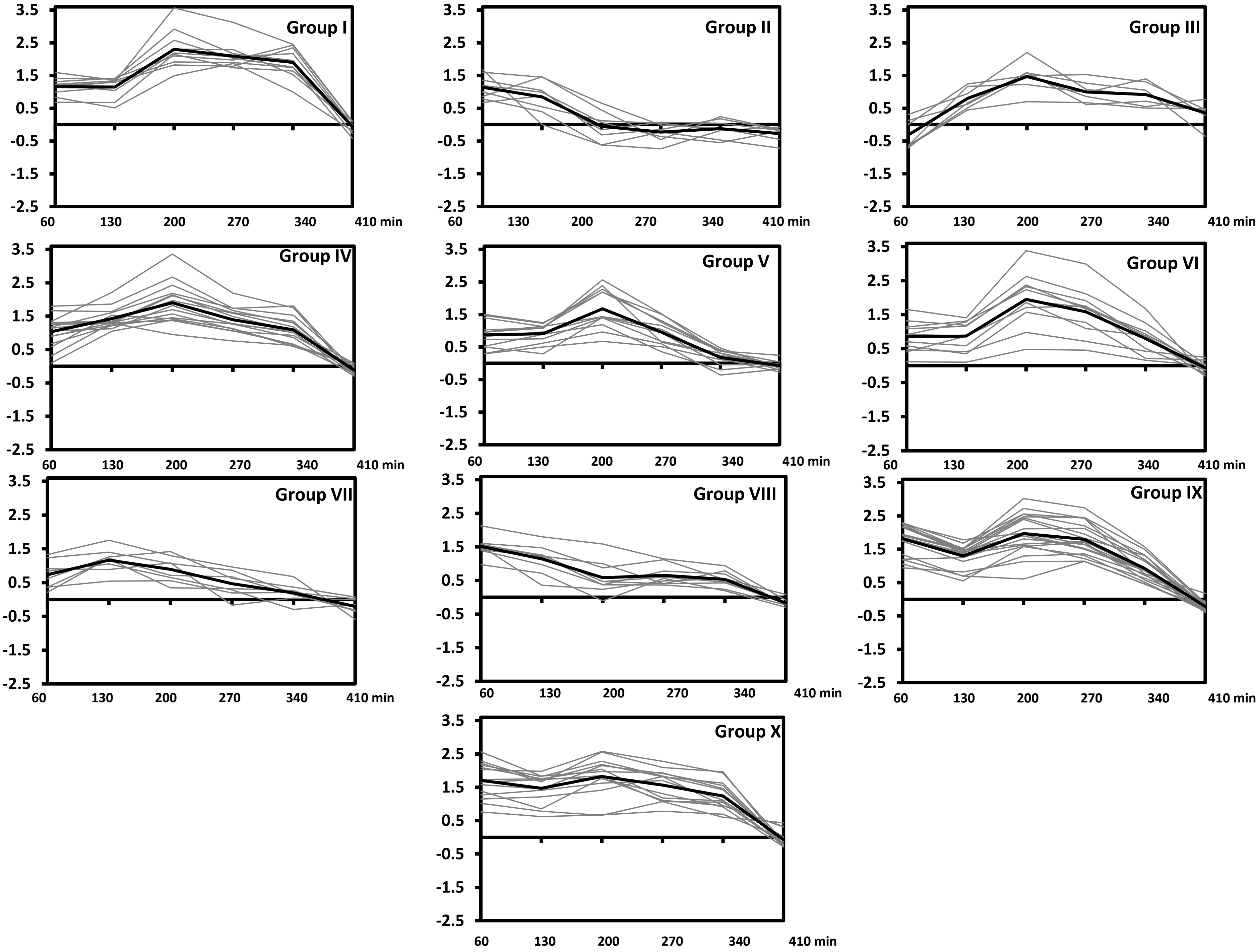
Group I
Group II
Group III
Group IV
Group V
Group VI
Group VII
Group VIII
Group IX
Group X
3.5
2.5
1.5
0.5
-0.5
-1.5
-2.5
60
130
200
270
340
410 min
